# Resonant Varifocal Micromirror with Piezoresistive Focus Sensor

**DOI:** 10.3390/mi7040057

**Published:** 2016-03-30

**Authors:** Kenta Nakazawa, Takashi Sasaki, Hiromasa Furuta, Jiro Kamiya, Hideki Sasaki, Toshikazu Kamiya, Kazuhiro Hane

**Affiliations:** 1 Department of Nanomechanics, Tohoku University, Aramaki-aza Aoba 6-6-01, Aoba-ku, Sendai 980-8579, Japan; t_sasaki@hane.mech.tohoku.ac.jp (T.S.); hane2@hane.mech.tohoku.ac.jp (K.H.); 2 Research & Development Center, Panasonic Industrial Devices SUNX Co., Ltd.; Ushiyama-cho 2431-1, Kasugai 486-0901, Japan; furuta.hiromasa@jp.panasonic.com (H.F.); kamiya.jiro@jp.panasonic.com (J.K.); sasaki.hsx@jp.panasonic.com (H.S.); kamiya.toshikazu@jp.panasonic.com (T.K.)

**Keywords:** varifocal mirror, piezoresistor, resonant vibration, microelectromechanical systems

## Abstract

This paper reports a microelectromechanical systems (MEMS) resonant varifocal mirror integrated with piezoresistive focus sensor. The varifocal mirror is driven electrostatically at a resonant frequency of a mirror plate to obtain the wide scanning range of a focal length. A piezoresistor is used to monitor the focal length of the varifocal mirror. The device is made of a silicon-on-insulator (SOI) wafer and a glass wafer. A mirror plate and a counter electrode are fabricated by a top silicon layer of the SOI wafer and on the glass wafer, respectively. The piezoresistor is fabricated by ion implantation on a supporting beam of the mirror plate. The stress variation of the beam, which is detected by the piezoresistor, correspond the focal length of the varifocal mirror. The focus length varies from −41 to 35 mm at the resonant frequency of 9.5 kHz. The focal length of the varifocal mirror is monitored by the piezoresistor in real time.

## 1. Introduction

The Varifocal mirror is a key optical component for adjustment of focal length. The varifocal mirrors based on the microelectromechanical systems (MEMS) technology realize miniaturization of optical systems, high speed focusing and low energy consumption. The varifocal mirrors have many applications for laser measurement systems and imaging systems such as confocal laser scanning microscopy, which needs the scanning frequency of 4 kHz [1] and multi-focus imaging, which needs the scanning frequency of 12 kHz [2]. For those applications, high-speed scanning, wide-range focus adjustment with low aberration is required. The several varifocal mirrors which use the different membrane materials (e.g., silicon nitride, silicon and polymer [3,4]) and the different driving methods (e.g., electrostatic and electrothermal) were reported. Li *et al.* reported an electrothermally actuated bimorph varifocal mirror. The power consumption to change the radius of curvature from 22 to 28 mm was 33 mW [5,6]. Moghimi *et al.* reported an electrostatically actuated SU-8 membrane varifocal mirror, which had the vertical air channels for adjusting the air damping to realize high speed focus control [7]. Sasaki *et al.* reported an electrostatically actuated resonant varifocal mirror to decrease the driving voltage [8].

To measure the focal length of the varifocal mirror is important for the applications. The resonant frequency and the amplitude of the varifocal mirror are affected by the environment such as pressure and temperature [9,10]. Therefore, it is difficult to decide the focal length by the applied voltage and frequency. The focal length can be monitored precisely by an integrated focus sensor. The piezoresistor is suitable to integrate with the varifocal mirror because piezoresistor is reliable and high sensitivity. However, there are few reports on the integration of varifocal mirrors with focus sensors.

In this study, we present an electrostatically actuated resonant varifocal mirror with piezoresistor to monitor the focal length. The design and fabrication of the varifocal mirror with the focus sensor are described in Section 2 and Section 3. The mechanical and optical characterization of the varifocal mirror is described in Section 4.2. In Section 4.3, the focus length monitoring by the piezoresistor is demonstrated.

## 2. Principle and Design

### 2.1. Basic Structure

**Figure 1:**
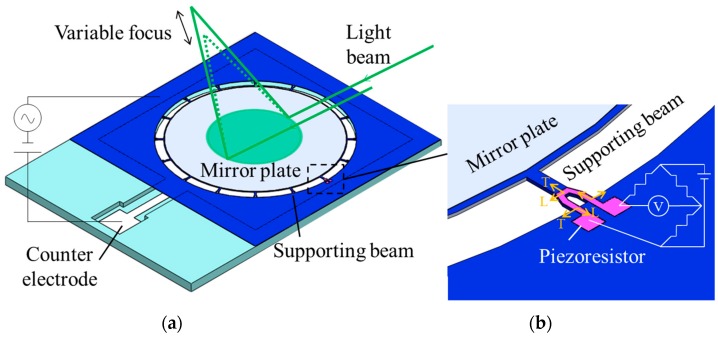
Schematic diagrams of the varifocal mirror with piezoresistive focus sensor. (**a**) Whole view of the varifocal mirror, (**b**) magnified of the piezoresistor part.

Figure 1 shows the schematic diagram of the proposed device. In Figure 1a, the device consists of the mirror plate and the counter electrode. The sixteen beams are used to suspend the mirror plate. The focal length is changed by deforming the mirror plate by electrostatic force. A piezoresistor is installed in the beam. The focal length of the varifocal mirror is obtained from the signal of the piezoresistor shown in Figure 1b, which is named “focus sensor” in this paper. The resistance of the piezoresistor changes by the strain caused by the mirror plate deformation. The strain corresponds to the focus length of the varifocal mirror.

### 2.2. Vibrationall Varifocusing

In order to drive the varifocal mirror, the mechanical resonant vibration is used in this experiment for enlarging the focus range [11]. The mirror plate of the varifocal mirror is oscillated in the lowest resonant mode of the circular plate. If the energy loss of the vibration is small, the vibrational deformation of the mirror plate is larger than that of the static deformation at the same magnitude of voltage. The enhancement of the deformation is more than one order of magnitude in vacuum since the air friction is the dominant loss for the vibration. The large deformation in the resonant vibration of the mirror plate is utilized for the variation of the focal length in this experiment. However, since the deformation is periodic, the stroboscopic illumination in phase with the periodic vibration is used for focusing.

**Figure 2:**
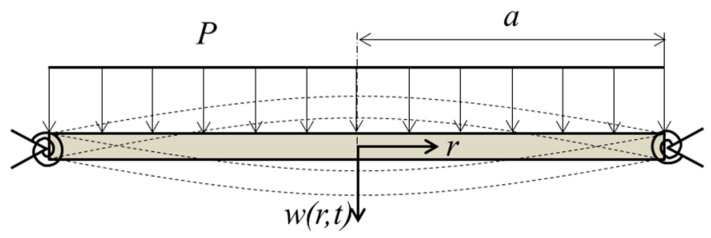
Theoretical vibration model of the varifocal mirror.

Since the instantaneous shape of the oscillating mirror is used for focusing, it is important to know the theoretical shape of the mirror deformation. Figure 2 shows the theoretical model of the varifocal mirror. The circular elastic varifocal mirror is supported by the springs at the circumference. The support condition of the actual mirror shown Figure 1 is softer than the fully clamped condition of the mirror plate and harder than the simply support condition. However, for simplicity, the supported conditions of the spring used in the model are assumed to be the fully clamped and the simply supported conditions. On the mirror surface, the periodical electrostatic force is applied. It is assumed that the force is uniformly distributed in the model for simplicity. The partial differential equation of the motion for the model of varifocal mirror is expressed as follows [12],

(1) $$\nabla w\left(r,t\right)+\frac{\rho }{D}\frac{\partial^{2}w\left(r,t\right)}{\partial t^{2}}=\frac{P\left(t\right)}{D}$$

here, *w* is the displacement and *r* is the radial position as shown in Figure 2. *t* is time, *ρ* is density and *ν* is Poisson’s ratio. *P* is the electrostatic force per the unit area. The flexural rigidity *D* is given by:

(2) $$D=\frac{Eh^{3}}{12\left(1-\nu^{2}\right)}$$

**Figure 3:**
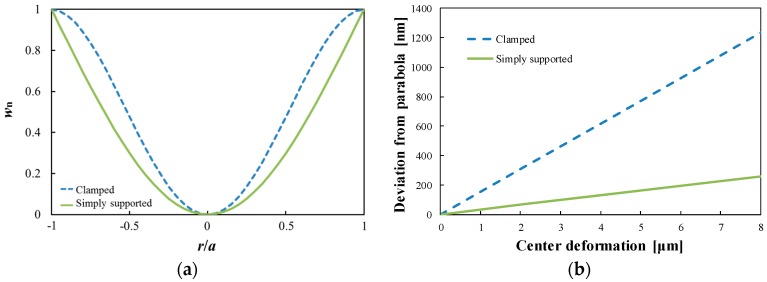
(**a**) Surface profiles of the circular plate at resonant frequency. (**b**) Comparison of the deviations from parabola between the clamped and simply supported conditions.

The displacement of the mirror is obtained by solving Equation (1) as follows:

(3) $$w\left(r,t\right)=a\left[I_{0}\left(\kappa_{1}\right)J_{0}\left(\kappa_{1}r/a\right)-J_{0}\left(\kappa_{1}\right)I_{0}\left(\kappa_{1}r/a\right)\right]g_{1}\left(t\right)$$

here, *a* is the radius of the mirror, *I*_0_ is the 0th order modified Bessel function, *J*_0_ is the 0th order Bessel function, *κ*_1_ is the eigenvalue. *g*_1_ is the term dependent on the loading function and the boundary condition, and expressed as follows:

(4) $$g_{1}\left(t\right)=\frac{a}{D}\sqrt{\frac{D}{m}}\frac{P}{\kappa_{1}^{3}}\frac{\left[I_{0}\left(\kappa_{1}\right)J_{1}\left(\kappa_{1}\right)-J_{0}\left(\kappa_{1}\right)I_{1}\left(\kappa_{1}\right)\right]}{N_{11}}\int_{0}^{t}\sin\left(t-\tau \right)\omega_{1}\tau d\tau$$

here, *N*_11_ is expressed as follows:

(5) $$N_{11}=\frac{1}{2}\left[I_{0}^{2}\left(\kappa_{1}\right)J_{1}^{2}\left(\kappa_{1}\right)-J_{0}^{2}\left(\kappa_{1}\right)I_{1}^{2}\left(\kappa_{1}\right)\right]-\frac{1+\nu +\beta a/D}{1-\nu -\beta a/D}I_{0}^{2}\left(\kappa_{1}\right)J_{0}^{2}\left(\kappa_{1}\right)$$

here, β is the edge spring constant. When the circular plate is simply supported, *κ*_1_ is equal to 2.1080 and β*a*/*D* + *ν* is 0. When the plate is clamped, *κ*_1_ is equal to 3.1962 and β*a*/*D* + *ν* is infinite. The actual varifocal mirror has the values of *κ*_1_ and β*a*/*D* + *ν* between those in the clamped and the simply supported conditions because the varifocal mirror plate is supported by the narrow beams. We calculated the normalized displacement *w*_n_ for the two cases as a function of the dimensionless radius *r*/*a* using the parameters; *a* = 1 mm, *h* = 5 μm, *E* = 169 GPa, *ν* = 0.28, which are shown in Figure 3a. The slope of the plate at the circumference in the simply supported condition is larger than that in the clamped condition. On the other hand, the slope of the plate at *r*/*a* = 0.5 in the simply supported condition is smaller than that in the clamped condition. From the calculated displacements, the maximum deviations from a parabola are shown in Figure 3b as a function of the displacement of plate at the center. The deviation from the parabola is proportional to the center deformation and smaller when the mirror circumference is simply supported. In the actual design, the deviation from the parabola is considered to be between those in the clamed and the simply supported conditions.

### 2.3. Piezoresistor

The fractional change of the resistance Δ*R* of the piezoresistor is expressed as follows [13]:

(6) $$\frac{\Delta R}{R}=\pi_{L}\sigma_{L}-\pi_{T}\sigma_{T}$$

(7) $$\pi_{L}=\pi_{11}+2\left(\pi_{44}+\pi_{12}-\pi_{11}\right)\left(l_{1}^{2}m_{1}^{2}+l_{1}^{2}n_{1}^{2}+m_{1}^{2}n_{1}^{2}\right)$$

(8) $$\pi_{T}=\pi_{12}+2\left(\pi_{44}+\pi_{12}-\pi_{11}\right)\left(l_{1}^{2}l_{2}^{2}+m_{1}^{2}m_{2}^{2}+n_{1}^{2}n_{2}^{2}\right)$$

where *R* is the resistance, π is the piezoresistive coefficient, σ is the applied stress and *l*, *m*, *n* are the direction cosines. The subscript L and T show longitudinal and transverse directions for the electric current of the piezoresistor as shown in Figure 1b. To increase the fractional change of the resistance, an appropriate piezoresistive coefficient should be chosen. As well as the crystal orientation, the piezoresistive coefficient is known to depend on the resistivity and the doping type. The p-type silicon is used as the piezoresistor. The maximum piezoresistive coefficient of the p-type (100) single crystalline silicon is obtained by fabricating the piezoresistor along <110> direction [14]. When the piezoresistor is fabricated along <110> direction, the Equation (6) is simplified as follows,

(9) $$\frac{\Delta R}{R}=\frac{1}{2}\left(\sigma_{L}-\sigma_{T}\right)\pi_{44}$$

Since the maximum applied stress is generated at the fixed end of beams, the piezoresistor is formed there along <110> direction. The U shaped piezoresistor is formed for obtaining twice higher sensitivity. Although one end of the beam is divided into two parts, as a simplified model of the varifocal mirror, the stress distribution on the beam is roughly estimated from a straight beam. The stress σ_b_ in the longitudinal direction at fixed end of the beam is given by:

(10) $$\sigma_{b}=\frac{4E_{b}h_{b}}{a^{2}}w$$

where *E*_b_ and *h*_b_ are the Young’s modulus and the thickness, respectively.

### 2.4. Design Details

**Figure 4:**
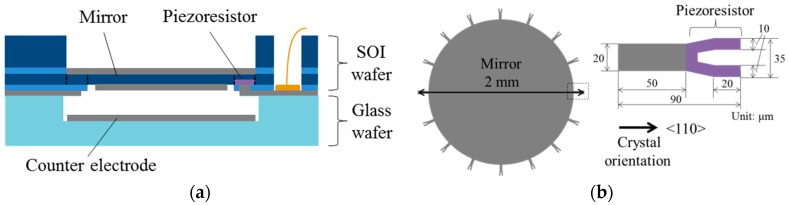
(**a**) Cross-sectional schematic diagram of the varifocal mirror. (**b**) Schematic diagram of the varifocal mirror plate and piezoresistor.

Figure 4a shows the cross sectional schematic diagram of the varifocal mirror. Figure 4b is the mirror part and the piezoresistor part. The varifocal mirror is driven by the electrostatic force generated by the counter electrode in Figure 4a. The varifocal mirror is made of a SOI wafer and a glass wafer (Pyrex glass). The n-type (100) top silicon layer of the SOI wafer is used as the mirror plate. The mirror diameter and thickness are 2 mm and 5 μm, respectively. The gap between the mirror plate and the counter electrode is 25 μm, which is fabricated by etching the glass wafer. The counter electrode is formed on the glass wafer. The mirror circumference is supported by 16 beams to make the support which is close to the simply supported boundary condition in order to minimize the deviation from parabola. The beam is Y shape of 90 μm in length and 20 μm in width. The spring constant of the circumference decreases by 95% comparing with the case that the mirror plate is directly fixed at the circumference. In order to calculate the resonant frequency, finite element method (COMSOL Multiphysics 4.4, COMSOL, Inc., Burlington, MA, USA) is used because the structure of the varifocal mirror is complex. The dimension of the structural model is based on the designed device shown in Figure 4b. The tips of the beam are fixed. For the material property of the single crystal silicon, we referred to [14]. The piezoresistor is formed on the U shape part of the Y shaped beam. By substituting *E*_b_ = 169 GPa, *h*_b_ = 5 μm, *a* = 1 mm and *y*_c_ = 7 μm into Equation (10), σ_b_ = 23.7 MPa is obtained. Since the piezoresistive coefficient of p-type silicon (7.8 Ω·cm) π_44_ is 138.1 × 10^−11^ Pa^−1^ [13], the fractional change of 1.6% is obtained by substituting σ_b_ and π_44_ into Equation (9). The holes on mirror for decreasing air damping are not formed to prevent from degrading optical reflection performance.

## 3. Fabrication and Measurement Setup

**Figure 5:**
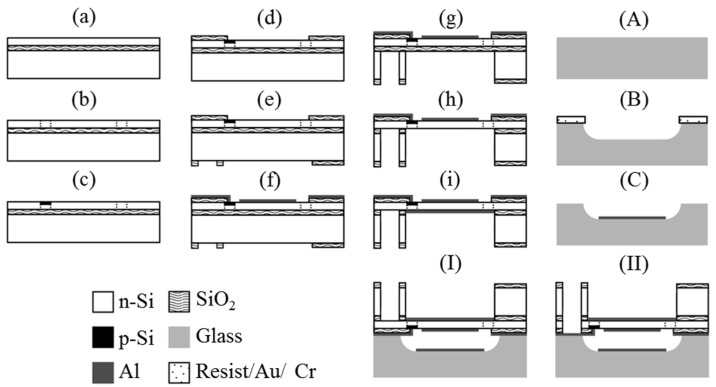
Process steps for the fabrication of the varifocal mirror. (**a**) SOI wafer; (**b**) Top Si etching; (**c**) B ion implantation; (**d**) Annealing, oxidation and buffered hydrofluoric acid (BHF) etching; (**e**) SiO_2_ deposition and BHF etching; (**f**) Al deposition and etching; (**g**) Handle Si etching; (**h**) BOX etching; (**i**) Al deposition; (**A**) Glass wafer; (**B**) HF etching with resist polymer/Au/Cr mask; (**C**) Al deposition and etching; (**I**) Anodic bonding; (**II**) Top Si and SiO_2_ etching for the electrode pad of the piezoresistor.

Figure 5 shows the fabrication processes. A SOI wafer is used for the varifocal mirror plate. The thicknesses of the top silicon layer, the buried oxide layer and the handle silicon layer are 5, 2 and 200 μm, respectively. The resistivity of the top layer silicon is 1–5 Ω·cm and the doping type is n-type. The mirror and the sensor are fabricated on the (100) top silicon layer. First, the top silicon layer is etched to form the beam structure (b). Next, boron ions are implanted (B^+^ at 35 keV, 9 × 10^14^ atoms/cm^2^) to fabricate the piezoresistors (c). Then the wafer is annealed at 1100 °C for activating the implanted boron ions in nitrogen environment. The top layer is oxidized and etched to form the contact holes for sensor by buffered hydrofluoric acid (BHF) (d). The silicon dioxide layer is deposited and patterned to form the hard etching mask of the silicon handle layer (e). The aluminum film is evaporated on the top layer and etched by tetramethylammonium hydroxide (TMAH) 2.38% solution to form wiring for the sensor. Then, the wafer is annealed at 420 °C for forming ohmic contacts (f). Next, the silicon handle layer is removed by deep reactive ion etching (g). Then, the buried oxide layer is removed by BHF (h). The 100 nm thickness aluminum is formed on the mirror surface as a reflective film with stencil mask (i). The calculated reflectivity is 93% at visible wavelength. The surface roughness is equal to that of the top silicon layer of the SOI wafer, which is a few nanometers. The flatness of the mirror is measured without applying voltage as shown later. The stencil mask is useful for patterning on the free standing structures. The mask is also used for the sensor electrodes. The counter electrode is fabricated on a 300 μm thick glass wafer (Pyrex glass). A resist polymer/gold/chromium film is used as the mask for etching the glass substrate with 49% hydrofluoric acid. A 25 μm gap between mirror and electrode is formed (B). An aluminum film is deposited on the glass wafer and patterned by TMAH2.38% solution with a polymer mask (C). The Al film is used for the counter electrode and is connected through a channel shown in Figure 1a to an electrode pad. The glass substrate is bonded to the top aluminum layer of the SOI wafer by anodic bonding (I). The bonding is carried out at 400 °C and 300 V. Then, the silicon and silicon dioxide layers are removed to make electric wiring to the sensor electrodes (II).

**Figure 6:**
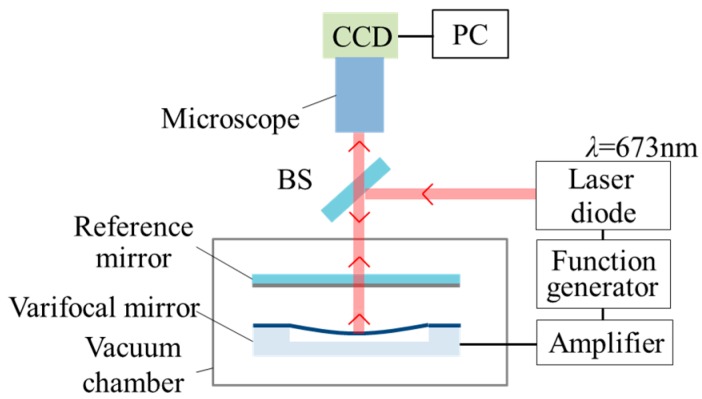
Stroboscopic Fizeau interferometer for the measurement of the deformation of the varifocal mirror. CCD: Charge-coupled device camera; BS: Beam splitter; PC: Personal computer.

The stroboscopic Fizeau interferometer is used to measure the dynamic surface profile of the varifocal mirror. Figure 6 shows the measurement setup. The wavelength of the laser source is 673 nm. The laser pulse width is 5% of the period of the varifocal mirror vibration. When the mirror is at the maximum deformation, the laser pulse width causes 0.02% measurement error of the deformation, which means that the laser pulse width does not affect the deformation measurement very much because the mirror velocity is close to zero under the condition. The measurement is carried out in a vacuum chamber to decrease the squeeze damping. The pressure in the vacuum chamber is 3 Pa. The sinusoidal voltage *V*_PP_ (peak-to-peak voltage) with a DC offset voltage *V*_DC_ is applied for driving the varifocal mirror. The laser Doppler vibrometer is used to measure the relationship of the dynamic deformation of the varifocal mirror.

## 4. Results and Discussion

### 4.1. Fabrication Results

**Figure 7:**
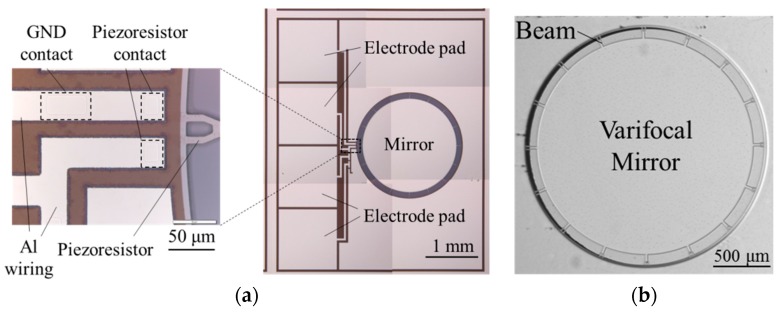
(**a**) Optical micrographs of the fabricated varifocal mirror and the piezoresistor with wiring. (**b**) The refraction surface of the varifocal mirror. GND: Ground.

Figure 7a shows the optical micrograph of the sensor part and the mirror on the top layer silicon. The piezoresistors are connected to form Wheatstone bridge. The optical micrograph of the reflection surface of the varifocal is shown in Figure 7b.

### 4.2. Mirror Characteristics

**Figure 8:**
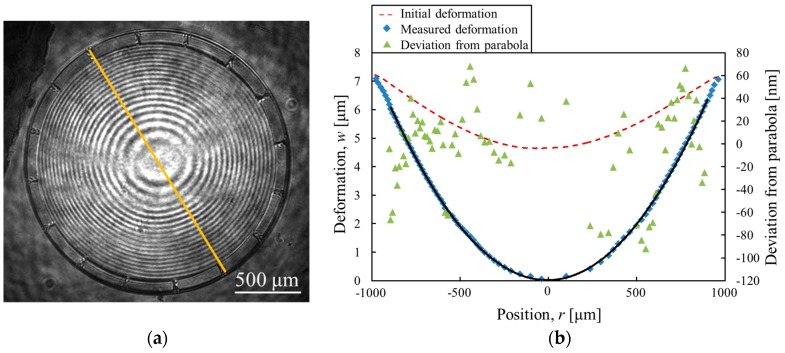
(**a**) Interference image and (**b**) surface profile of the varifocal mirror.

For the optical systems, the high surface precision of the varifocal mirror is required. To estimate the surface precision, the surface profile of the mirror is measured. Figure 8a shows the interference image from the stroboscopic interferometer at maximum oscillation amplitude at the voltage of 15 *V*_DC_ + 30 *V*_PP_ and the frequency of 9.5 kHz. The clear concentric fringes are seen in Figure 8a. In Figure 8b, the deformation of the mirror along the line shown in Figure 8a is plotted. The fitting curve is given by an equation *w* = 7.71 × 10^−6^*r*^2^ + 8.35 × 10^−5^*r* + 1.83 × 10^−2^, where *w* (in micrometers) is the deformation as a function of the radius *r* (in micrometers). The initial deformation of the mirror plate is also measured by a white light interferometer (MSA500, Polytec GmbH, Waldbronn, Germany) as shown in Figure 8b. The center deformation of the varifocal mirror is 7.3 μm in this condition. The deviation from the fitted parabola in the region |*r*| < 0.8*a* is also shown in Figure 8b. The deviation from the parabola is within 150 nm. Considering the He-Ne laser wavelength (λ_He-Ne_ = 633 nm), the surface precision less than λ_He-Ne_/4 is obtained. From the theoretical analysis shown in Figure 3, the deviation from parabola is within 240 nm at the center deformation of 7.3 μm in the simply supported condition. The mirror surface precision of the experimental results is a little better than the theoretical results since the initial deformation may affects the dynamical surface profile.

**Figure 9:**
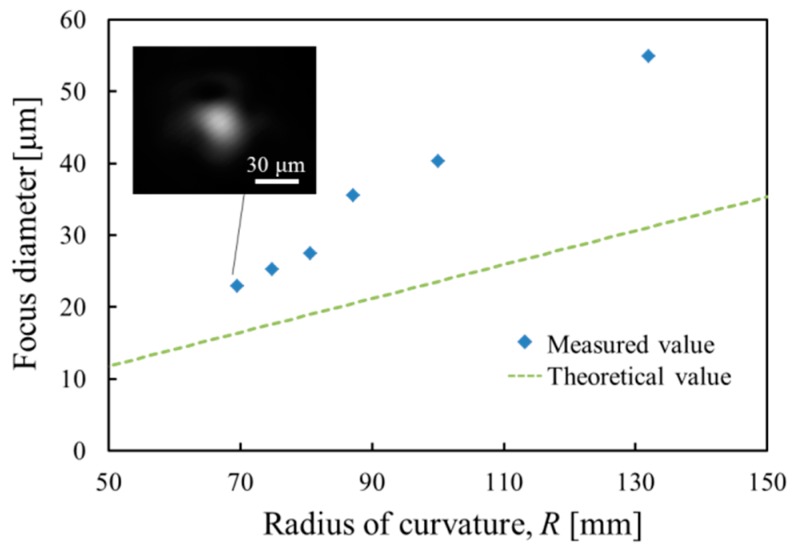
Focus spot diameter as a function of the radius of curvature of the varifocal mirror and the spot image at a radius of curvature of 70 mm.

To investigate the focusing function of the varifocal mirror, the focus spot diameter measured as a function of the radius of curvature of the varifocal mirror is shown in Figure 9. The focus diameter correlates with the image resolution obtained from an instrument using the varifocal mirror. The varifocal mirror is driven at the voltage of 15 *V*_DC_ + 30 *V*_PP_ and the frequency of 9.5 kHz. To change the focus, the phase of the laser pulse is changed. The focus spot is obtained by using a charge-coupled device (CCD) camera (UI-2240SE, IDS Imaging Development Systems GmbH, Obersulm, Germany). The intensity distribution of the focus spot is measured using the image analysis software. The theoretical focus diameter *D*_f_ is also plotted in Figure 9, which is defined by full-width-at-half-maximum intensity and expressed as follows [15],

(11) $$D_{f}=0.76\frac{\lambda R_{c}}{D_{\text{in}}}$$

here, λ is the wavelength of the laser light for measurement, *R*_c_ is the radius of mirror curvature, *D*_in_ is the diameter of the incident collimated beam. The value of *D*_f_ corresponds to the diffraction limited value. In Figure 9, the dotted line shows the diffraction limit obtained by Equation (11). The deviation of the measured value from the theoretical value is considered to be caused by the non-parabolic surface profile and the measurement error by laser pulse width. The finite pulse width of the laser irradiation generates the error of measured spot size. For example, because the mirror velocity is nearly zero at the radius of the radius of the curvature of 70 mm, the measurement error by the mirror velocity is negligible as mentioned in Section 3. The laser spot size enlargement due to the non-parabolic surface profile is estimated to be 30%. At the radius of the curvature of 130 mm, the spot size enlargement is 43%. Because the mirror velocity is not zero, the spot size is enlarged by not only the non-parabolic surface profile but also the measurement error by laser pulse width. The latter is calculated to be 28% and the former is estimated to be 15%.

### 4.3. Focus Sensor Signal

**Figure 10:**
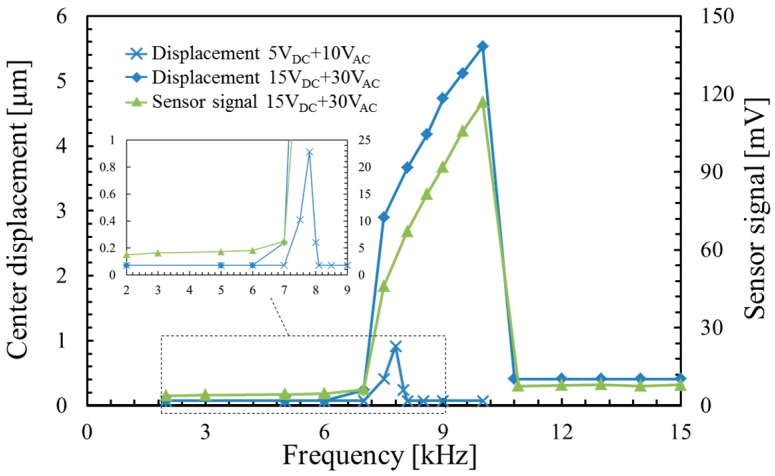
Frequency response of the deformation of the varifocal mirror and the piezoresistor.

Figure 10 shows the frequency responses of the center displacement of the varifocal mirror and the piezoresistor signal voltage. The sensor signal voltage is given by the peak-to-peak value. The varifocal mirror is driven at the voltage of 15 *V*_DC_ + 30 *V*_PP_ and 5 *V*_DC_ + 10 *V*_PP_. The Wheatstone bridge is driven at the voltage of 1.5 V. At the voltage of 5 *V*_DC_ + 10 *V*_PP_, the resonant frequency is 7.8 kHz and the quality factor is 22. In our measurements, the distribution of the resonant frequency for each device is about 1 kHz in the same wafer. The time constant is decided by the focusing, which is approximately 0.1 μs. The resonant frequency is smaller than that obtained by FEM analysis (11.1 kHz). The difference between the measured and the calculated resonant frequency can be attributed to a compressive stress acting on the mirror plate. The deposited aluminum film and the anodic bonding may generate the compressive stress. At the voltage of 15 *V*_DC_ + 30 *V*_PP_, the mirror amplitude increases in the frequency range between 7.5 and 10 kHz. The center displacement at a frequency of 10 kHz is 92 times larger than that in frequency range from 2 to 6 kHz. The piezoresistor signal agrees well with the center displacement of the varifocal mirror. The nonlinear phenomenon is described as follows. At a small voltage of 5 *V*_DC_ + 10 *V*_AC_, although the oscillation curve is slightly asymmetric, it has a peak at 7.8 kHz and does not have the sudden transition observed at the higher voltage as shown in the inset of Figure 10. Therefore, spring hardening effect is small under the condition. Increasing the applied voltage, the frequency at the peak of the oscillation curve shifts with the increase of the amplitude. The resonant frequency at the voltage of 15 *V*_DC_ + 30 *V*_AC_ is larger by a factor of 6 than that at the voltage of 5 *V*_DC_ + 10 *V*_AC_. However, the sudden transition from the peak at the frequency of 10 kHz to the amplitude of 0.41 μm (1/11 of the peak value) is observed at the high voltage. The sudden transition has a hysteresis when the applied voltage is decreased. This phenomenon is explained by the oscillation curve of the spring hardening effect [16]. The resonant frequency which is corresponding to the frequency at the peak increases because the stress caused by the elongation of the mirror plate increases the stiffness varifocal mirror. Therefore, the resonant frequency of the varifocal mirror depends on the displacement.

In order to estimate the spring hardening effect, a doubly clamped beam is considered as a model of the varifocal mirror for simplicity. For the beam analysis, only longitudinal stress is considered. On the other hand, for the circular plate analysis, the circumferential stress σ_t_ and the radial stress σ_r_ are considered. In case of *r* = 0, *a*/2 and *a*, the ratio σ_r_/σ_t_ are equal to 1, 0.5 and 3, respectively [17]. When the circumferential stress is ignored, the circular plate can be assumed to be the beam. The difference of the stress caused by the spring hardening effect between the circular plate and the beam is estimated to be a few hundred percent by the ratio σ_r_/σ_t_. Therefore, the doubly-clamped beam can estimate in the same digit. The spring constant of the doubly clamped beam *k*_N_ is expressed as follows when a compressive or tensile stress is applied [18,19]:

(12) $$k_{\text{NC}}=\frac{\sigma_{N}bh_{b}}{a\left[\left\{1-\cos\left(j_{0}a/2\right)\right\}/\left\{2j_{0}a\sin\left(j_{0}a/2\right)\right\}-1/8\right]}\;(\sigma_{N}\;<\;0)$$

(13) $$k_{\text{NT}}=\frac{4\sigma_{N}bh_{b}}{a-2\left\{\cosh\left(j_{0}a\right)-1\right\}/j_{0}\sinh\left(j_{0}a\right)}\;(\sigma_{N}\;>\;0)$$

here, σ_N_ and *b* are the stress and the width of the beam, respectively. Here, *j*_0_ is given by:

(14) $$j_{0}=\sqrt{\frac{12\sigma_{N}}{E_{b}h_{b}^{2}}}$$

On the other hand, the resonant frequency *f*_r_ of the clamped beam is given by:

(15) $$f_{r}=\frac{1}{2\pi }\sqrt{\frac{k_{N}}{m}}$$

where *m* is the mass of beam. Using Equations (12)–(14), the stress σ_N_ of the spring hardening effect can be roughly estimated. The value of the resonant frequency in the small amplitude is obtained from the peak value of the frequency response at 5 *V*_DC_ + 10 *V*_PP_ in Figure 10 to be 7.8 kHz. Similarly, the value of the resonant frequency in large amplitude is estimated from the peak value at 15 *V*_DC_ + 30 *V*_PP_ in Figure 10 to be 10 kHz. The frequency increase from 7.8 to 10 kHz is considered to be changed the stress σ_N_ of the spring hardening effect. The stress sift is roughly estimated to be 1.7 MPa.

**Figure 11:**
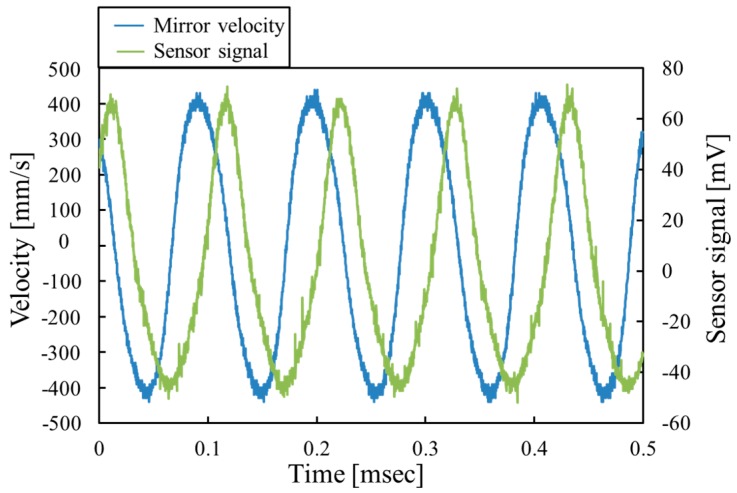
Velocity of the varifocal mirror at the center and the piezoresistor signal as a function of time.

Figure 11 shows the time dependence of the mirror velocity at the center of mirror and the piezoresistor signal at the applied voltage of 15 *V*_DC_ + 30 *V*_PP_ and the frequency of 9.5 kHz. The velocity of the varifocal mirror is measured by the laser Doppler vibrometer (MSA500, Polytec GmbH). The surface profile of the mirror plate is concave when the piezoresistor signal voltage is negative. Figure 11 shows that the varifocal mirror deforms sinusoidally. The signal of the piezoresistor is also periodical and the frequency agrees with that of the mirror velocity. However, the piezoresistor signal varies from −45 to 65 mV and is not completely a pure sinusoidal curve. These facts may be caused by the stress σ_bN_ causing the spring hardening effect. The stress from the bending σ_b_ is calculated to be 24.7 MPa by Equation (10). The stress σ_bN_ causing the nonlinear effect is estimated to be 1.7 MPa from the resonant frequency shift as described above. The stress σ_bN_ affects the piezoresistor signal because the ratio σ_bN_/σ_b_ is 0.07. A compressive stress σ_b_ is always applied to the piezoresistor when the mirror shape is concave. In addition, the tensile stress σ_bN_ caused by the spring hardening effect also acts on the piezoresistor, which compensates the compressive stress σ_b_. Similarly, when mirror shape is convex, the tensile stress σ_b_ caused by the bending of mirror and the tensile stress σ_bN_ by the spring hardening effect are applied to the piezoresistor. Therefore, the amplitude of the piezoresistor signal voltage decreases when the mirror shape is concave, and, the amplitude increases when the mirror shape is convex. The above discussion can explain the waveform of the sensor signal shown in Figure 11.

**Figure 12:**
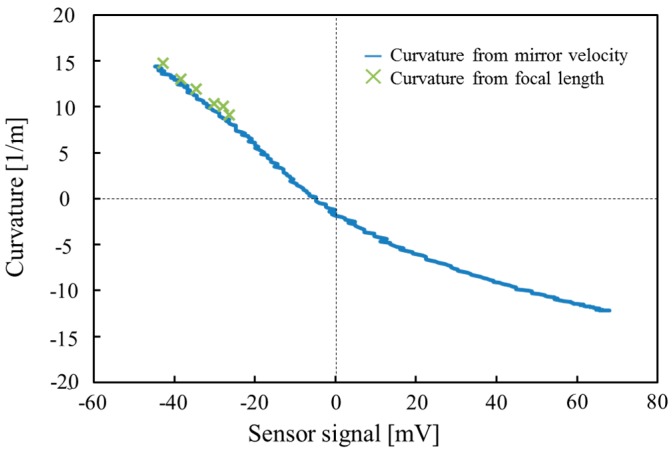
Curvature as a function of the piezoresistor signal.

For the actual use, it is important to know the relationship between the curvature of the varifocal mirror and the piezoresistor signal voltage. The curvature as a function of the piezoresistor signal voltage is shown in Figure 12. The varifocal mirror is driven at the voltage of 15 *V*_DC_ + 30 *V*_PP_ and the frequency of 9.5 kHz. The curvature is measured by the two methods. One is by a focal length measurement from the spot size and the other is the measurement of the mirror velocity shown in Figure 11. The cross mark in Figure 12 shows the result of the focal length measurement and the solid curve shows the result obtained from Figure 11. In the focal length measurement, the minimum spot position is measured by changing the distance of the image sensor. In the case of the focal length measurement, the curvature is measured only from 9 to 14 m^−1^ due to the limitation of the measurement setup. In the case of the mirror velocity measurement, the mirror velocity is assumed to be sinusoidal and the velocity signal is integrated to obtain the displacement of the varifocal mirror at the center position. Then, the displacement is converted to the curvature assuming the deformed shape is parabolic. The curvature of the varifocal mirror obtained from piezoresistor is in the range from −12.2 to 14.4 m^−1^, which correspond to the focal lengths from −41 to 35 mm. The result obtained from the mirror velocity agrees with that of the focal length measurement as shown in Figure 12. In Figure 12, the curvature as a function of the piezoresistor signal voltage is not linear as described in the explanation of the waveform in Figure 11. The nonlinear dependence can be compensated by software when the varifocal mirror is used in application systems. For actual use of the varifocal mirror, the focal length is obtained by the curvature as a function of the piezoresistor signal voltage as shown in Figure 12. Since the focal length is the inverse of the curvature, the piezoresistor signal can be used as a signal of the focus sensor. From Figure 12, in the signal region higher than −5 mV, the curvature is negative and the mirror is convex. In this region, the parallel beam is reflected by the mirror and diverges from the mirror. At the sensor voltage of −5 mV, the beam is reflected as it is since the curvature is zero. At the sensor voltage of 65 mV, the laser beam diverges from a virtual image focus at a focal length of −41 mm. In the sensor signal region lower than −5 mV, the curvature is positive and mirror is concave. The parallel laser beam converges on a real focus. At the sensor voltage of −45 mV, the curvature is 14.4 m^−1^, which is equal to the real focal length of 35 mm.

## 5. Conclusions

The electrostatically actuated resonant varifocal mirror integrated with the piezoresistive focus sensor was designed, fabricated and characterized. The top silicon layer of the SOI wafer was used as the mirror plate. The diameter of the mirror was 2 mm and the thickness was 5 μm. The piezoresistor was fabricated on the supporting beam by the ion implantation. The frequency response, the surface profile at the maximum amplitude, the focus diameter, the time dependence of the piezoresistor signal and the mirror velocity and the curvature of the varifocal mirror as a function of the piezoresistor signal were measured. The center deformation of the varifocal mirror was 7.3 μm at the voltage of 15 *V*_DC_ + 30 *V*_PP_ and the frequency of 9.5 kHz. The curvature of the varifocal mirror was obtained by the piezoresistor from −12.2 to 14.4 m^−1^, which corresponds to the focal lengths from −41 to 35 mm. The proposed varifocal mirror can be used for high speed focusing while monitoring the focal length. Therefore, this device could contribute to the development of innovative optical systems in the future.
